# Kartogenin Improves Doxorubicin-Induced Cardiotoxicity by Alleviating Oxidative Stress and Protecting Mitochondria

**DOI:** 10.3390/ijms26062434

**Published:** 2025-03-08

**Authors:** Hua Zhang, Yunpeng Wang, Rui Wang, Qin Yi, Hao Xu, Bin Tan, Jing Zhu

**Affiliations:** 1 Department of Pediatric Research Institute, National Clinical Research Center for Child Health and Disorders, Ministry of Education Key Laboratory of Child Development and Disorders, Children’s Hospital of Chongqing Medical University, Chongqing 400014, China; 2 Key Laboratory of Children’s Vital Organ Development and Diseases of Chongqing Health Commission, Chongqing 400014, China; 3 Department of Clinical Laboratory, Children’s Hospital of Chongqing Medical University, Chongqing 400014, China

**Keywords:** kartogenin, doxorubicin, cardiotoxicity, mitochondria, oxidative stress

## Abstract

Doxorubicin (DOX) is a common antitumor drug in clinical practice, but its clinical use is limited due to its cardiotoxic side effects. Oxidative stress and mitochondrial damage are involved in DOX-induced cardiotoxicity (DIC). Kartogenin (KGN) has been shown to have a potent ability to resist oxidative stress and maintain mitochondrial homeostasis. But the impact of KGN on DIC has not been reported. This study explores the potential protective effect of KGN on DIC. The effect of KGN on DIC was studied by establishing in vivo and in vitro DIC models. KGN reduced DOX-induced cardiac insufficiency, myocardial injury, oxidative stress damage, and mitochondrial dysfunction. Through network pharmacology and RNA sequencing (RNA-seq), the mechanism of KGN anti-DIC was highly correlated with oxidative stress and mitochondria. These findings suggest that KGN is a valuable and promising strategy for the prevention of doxorubicin cardiotoxicity.

## 1. Introduction

Because of its great effectiveness, the common anthracycline doxorubicin (DOX) has emerged as a key component of clinical cancer treatment [1]. While demonstrating therapeutic efficacy across multiple cancer types, the clinical application of DOX faces significant limitations owing to its propensity to induce cardiac complications [2]. Patients’ lives are at risk since doxorubicin-induced cardiotoxicity (DIC) is typically irreversible and can result in clinical congestive heart failure [3]. Therefore, the prevention and treatment of DIC depend heavily on the investigation of the precise mechanisms and the creation of novel therapeutic targets.

Numerous biological processes are involved in the intricate mechanics of DIC [4]. Substantial evidence implicates oxidative stress as the principal contributor to the pathogenesis of DIC [5]. When the antioxidant and oxidative systems are out of balance, it can disrupt several signaling pathways and impact biological processes. This condition is known as oxidative stress [6]. DOX has been mechanistically linked to the accumulation of ROS and subsequent oxidative stress-mediated cellular damage [7,8]. The primary source of ROS generation in vivo, mitochondria, are also the subcellular organelles that DIC gradually damages the most severely [9,10]. DOX has been shown to induce both mitochondrial structural alterations and mitochondrial malfunction in cardiomyocytes [11,12]. Oxidative stress injury is made worse by mitochondrial malfunction, which also increases the generation of reactive ROS [13]. In conclusion, DIC may be effectively treated and prevented by maintaining normal mitochondrial levels and lowering oxidative stress.

Kartogenin (KGN), a small molecule compound, is a potent inducer that effectively promotes the differentiation of pluripotent mesenchymal stem cells in chondrocytes [14]. Recent studies reveal its antioxidant properties and mitochondrial protective effect. Experimental data from Wang’s group revealed that the KGN-mediated activation of Nrf2/TXNIP signaling augments redox homeostasis in mammalian cells [15]. Not coincidentally, Tian et al. showed that KGN treatment effectively up-regulated the expression of GPX1 and HO-1, which attenuated oxidative stress [16]. Wang et al. also showed that KGN has a protective effect on mitochondria, as evidenced by its capacity to increase the membrane potential of mitochondria, decrease mitochondrial swelling, and prevent the outer mitochondrial membrane from rupturing [15]. While previous investigations have extensively explored KGN’s capacity to induce stem cell differentiation, its potential protective mechanisms against DIC-associated oxidative stress and mitochondrial impairment remain poorly characterized. This study therefore seeks to determine whether KGN mitigates DIC progression through the modulation of redox balance and preservation of mitochondrial homeostasis.

Our experimental design incorporated both cell-based and animal models to simulate DIC progression. Using echocardiography and histopathological staining, murine cardiac function and myocardial damage were evaluated. The detection of lipid peroxidation products and antioxidant enzymes from cardiac tissues and cells also assisted in determining the degree of oxidative stress. In addition to assessing ROS levels within cells, mitochondrial morphology and membrane potential were also examined, which are indicators of mitochondrial state. Through network pharmacology and sequencing data, we suggest potential pathways that KGN attenuates DIC. These novel insights hold translational potential for innovating therapeutic approaches aimed at mitigating the cardiotoxicity associated with anticancer regimens.

## 2. Results

### 2.1. KGN Alleviated DOX-Induced Cardiotoxicity in Mice

**Figure 1:**
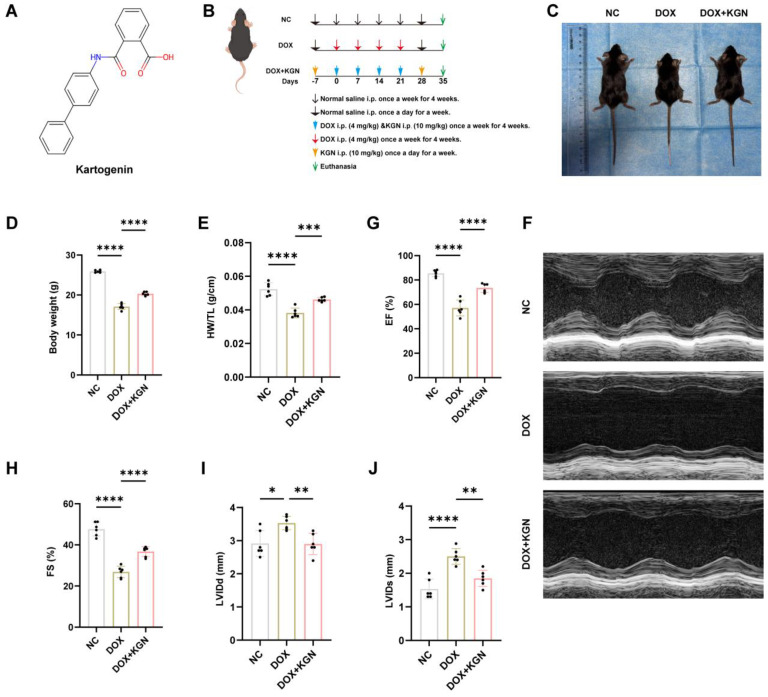
KGN alleviated DOX-induced cardiotoxicity in mice. (**A**) Chemical formula and two-dimensional structure of KGN. (**B**) Experiment timeline in vivo. (**C**) Representative images of body size. (**D**) Comparison of body weight among three groups of mice (*n* = 6). (**E**) HW/TL ratio among three groups of mice (*n* = 6). (**F**) Representative echocardiographic images. (**G**) Ejection fractions. (**H**) Fractional shortenings. (**I**,**J**) Measurements of end-systolic and diastolic left ventricular (LV) internal dimensions (*n* = 6). All data were expressed as the means ± SD. * *p* <0.05, ** *p* < 0.01, *** *p* < 0.001, **** *p* < 0.0001.

**Figure A1:**
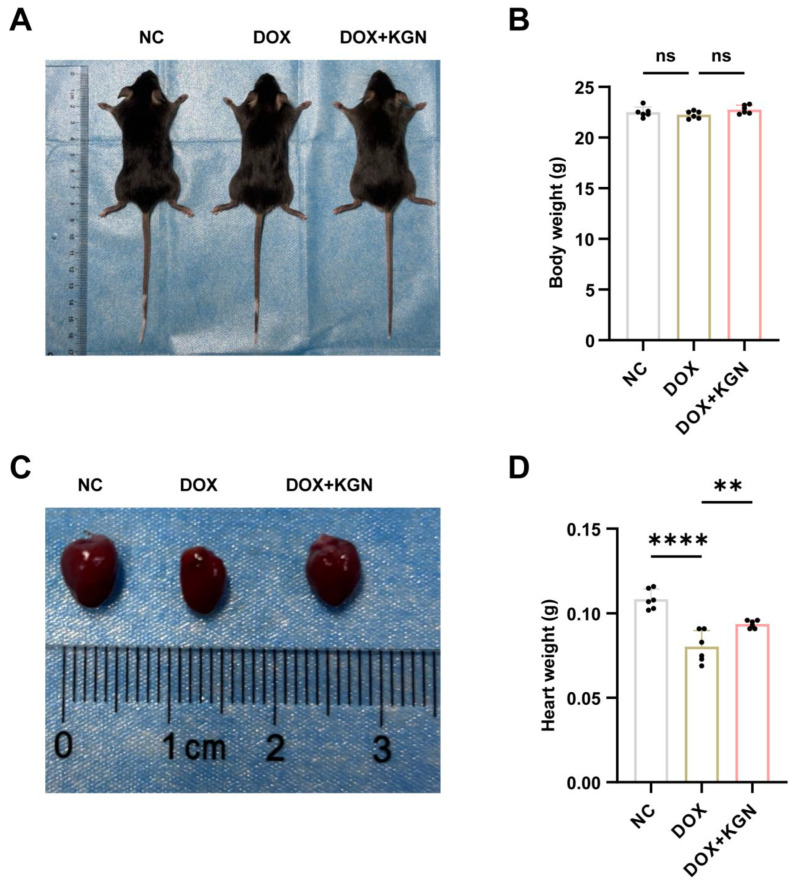
(**A**) Representative images of body size before modeling. (**B**) Mice body weight before modeling (*n* = 6). (**C**) Representative images of heart size after modeling. (**D**) Mice heart weight after modeling (*n* = 6). All data were expressed as the means ± SD. ** *p* < 0.01, **** *p* < 0.0001; ns means no significance.

To assess the cardioprotective effects of KGN in DIC, mice were pre-treated with KGN for 7 days prior to receiving intraperitoneal injections of DOX (4 mg/kg) weekly for 4 consecutive weeks. Following the DOX administration, KGN treatment continued for an additional 7 days. This approach allowed for the evaluation of both the prophylactic and therapeutic effects of KGN within the same group of mice (Figure 1A,B). Before the drug intervention, there was no difference in the size and weight of mice between the groups (Figure A1A,B). Interestingly, KGN pre-treatment and continued administration significantly prevented the DOX-induced reductions in body weight, heart weight, heart size and the HW/TL, as well as significantly improving cardiac function (Figure 1C–F and Figure A1C,D). DOX treatment led to notable cardiac dysfunction, characterized by increased LVIDd and LVIDs, as well as decreased EF and FS. Remarkably, KGN treatment significantly mitigated these functional impairments. The pre-treatment and continued administration of KGN resulted in reduced left ventricular internal diameter during diastole (LVIDd) and systole (LVIDs), and restored ejection fractions (EF) and fractional shortenings (FS), indicating enhanced ventricular performance and contractility (Figure 1G–I).

These findings demonstrate that KGN, through both its pre-treatment and continued post-DOX administration, provides substantial protection against DOX-induced myocardial dysfunction. Further research is warranted to explore the underlying mechanisms and the potential clinical applications of KGN in managing DOX-induced cardiotoxicity.

### 2.2. Histological and Biomarker Assessment of KGN’s Protective Effects on Doxorubicin-Induced Cardiotoxicity

To assess the histological effects of KGN on DIC, we performed HE and sirius red staining to evaluate myocardial structure and fibrosis.

**Figure 2:**
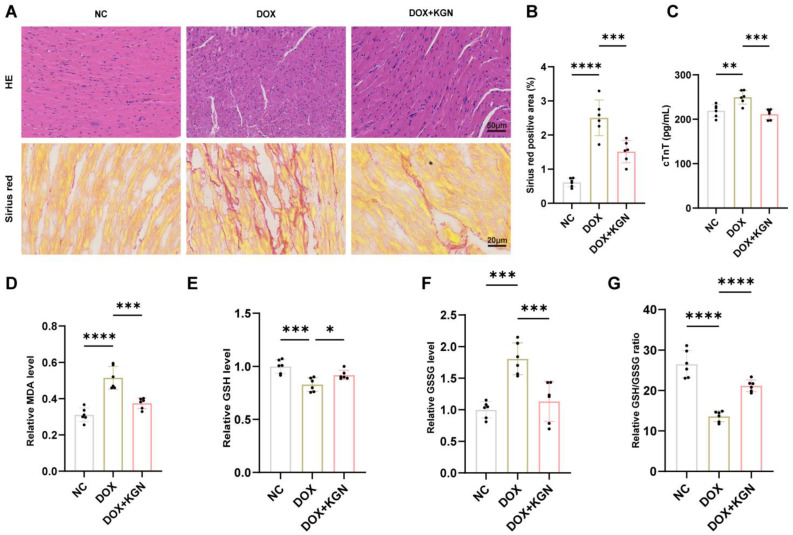
KGN protected myocardial tissue from DOX-induced damage and alleviated DOX-induced oxidative stress. (**A**) Representative hematoxylin–eosin (HE) staining and sirius red staining of myocardial vertical sections. (**B**) Quantitative analysis of sirius red stainings (*n* = 6). (**C**) Serum concentrations of cTnT (*n* = 6). (**D**–**G**) Relative levels of MDA, GSH, GSSG, and GSH/GSSG ratios in heart tissues (*n* = 6). All data were expressed as the means ± SD. * *p* <0.05, ** *p* < 0.01, *** *p* < 0.001, **** *p* < 0.0001.

The HE staining showed that the myocardial architecture in the control mice was dense and orderly. In contrast, DOX treatment caused a marked disarray of myocardial cells, with irregular and sparse arrangement, indicative of significant myocardial injury. KGN treatment significantly improved this pathological alteration, restoring myocardial cell alignment and density, and thus preserving structural integrity (Figure 2A).

Sirius red staining, which detects fibrosis, revealed extensive collagen deposition in DOX-treated mice, signifying pronounced myocardial fibrosis. KGN treatment significantly reduced collagen accumulation, indicating a substantial attenuation of fibrosis (Figure 2A,B).

We also measured cTnT levels, a marker of myocardial injury. Consistent with the histological results, DOX-treated mice exhibited elevated cTnT levels, which were significantly reduced by KGN treatment, further supporting its cardioprotective effects (Figure 2C).

Together, these findings demonstrate that KGN effectively alleviates both the structural and functional damage caused by DOX, suggesting its potential as a therapeutic agent for mitigating DOX-induced cardiotoxicity.

### 2.3. KGN Treatment Inhibited Myocardial Oxidative Stress in DOX-Treated Mice

Numerous studies have demonstrated the key role of oxidative stress in DOX-induced cardiotoxicity. To comprehensively evaluate the oxidative stress status in mouse models, we assessed several key biomarkers, including MDA, GSH, GSSG, and the GSH/GSSG ratio (Figure 2D–G). These indicators provide a thorough assessment of the oxidative damage and redox balance in myocardial tissue.

Our results demonstrated that DOX treatment led to a significant increase in MDA levels, a marker of lipid peroxidation, and a marked reduction in the GSH/GSSG ratio, reflecting oxidative damage and a disruption of redox homeostasis. Additionally, the GSSG levels were elevated, further supporting the presence of oxidative stress. Notably, KGN treatment significantly mitigated these oxidative alterations. It reduced the elevated MDA and GSSG levels induced by DOX, suggesting a reduction in lipid peroxidation and oxidative damage. Furthermore, KGN treatment restored the GSH/GSSG ratio, indicating that KGN helps to restore the balance between the reduced and oxidized forms of glutathione, a critical antioxidant system.

### 2.4. GO and KEGG Enrichment Analysis by Network Pharmacology and RNA-Seq

**Figure 3:**
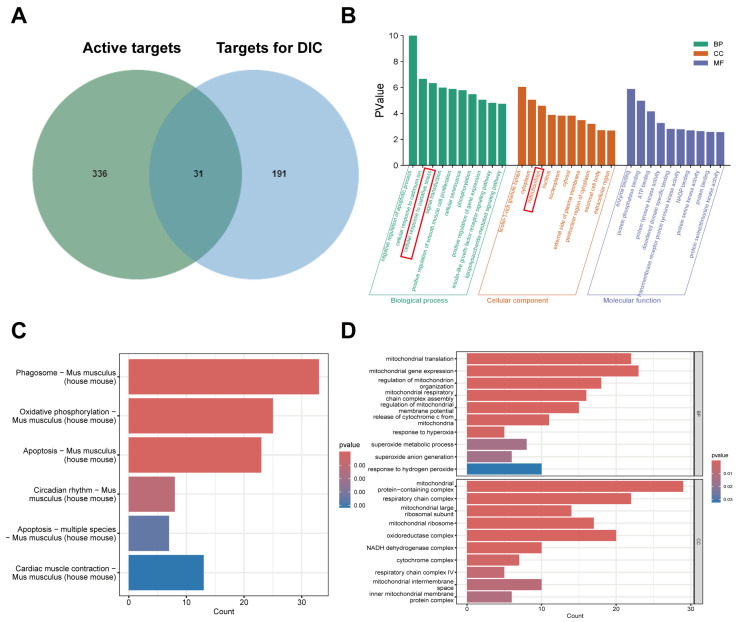
GO and KEGG enrichment analysis by network pharmacology and RNA sequencing. (**A**) Venn diagram of KGN active targets and DIC common targets. (**B**) GO analysis of the core targets of KGN against DIC. (**C**,**D**) KEGG and GO obtained by transcriptome sequencing.

After eliminating duplicate data, a total of 367 KGN active targets were identified from the PharmMapper and SwissTargetPrediction databases. In addition, a total of 222 DIC-related targets were identified from the GeneCards, and OMIM databases. A total of 31 common targets of KGN and DIC were identified (Figure 3A). The 31 common targets were submitted to the DAVID database for enrichment analysis with GO bioprocesses, and the results were sorted by *p*-value. The GO enrichment analysis produced a term related to 196 bioprocesses, 29 terms related to cellular components, and 39 terms related to molecular functions. Intersecting targets were significantly associated with cellular resistance to oxidative stress and mitochondria (Figure 3B). Meanwhile, three groups of mouse heart samples were subjected to RNA-seq and the results were taken from the intersections for GO and KEGG enrichment analyses. KEGG pathway analyses revealed that the oxidative phosphorylation pathway was significantly enriched in DOX-treated mice (Figure 3C). The GO enrichment results suggested that cellular constituents and bioprocesses were highly correlated with mitochondria (Figure 3D).

Our results draw the common conclusion from two different analytical approaches that the mechanism of KGN treatment of DIC is highly correlated with oxidative stress and mitochondria.

### 2.5. KGN Treatment Inhibited Myocardial Oxidative Stress in H9C2 Cells

**Figure 4:**
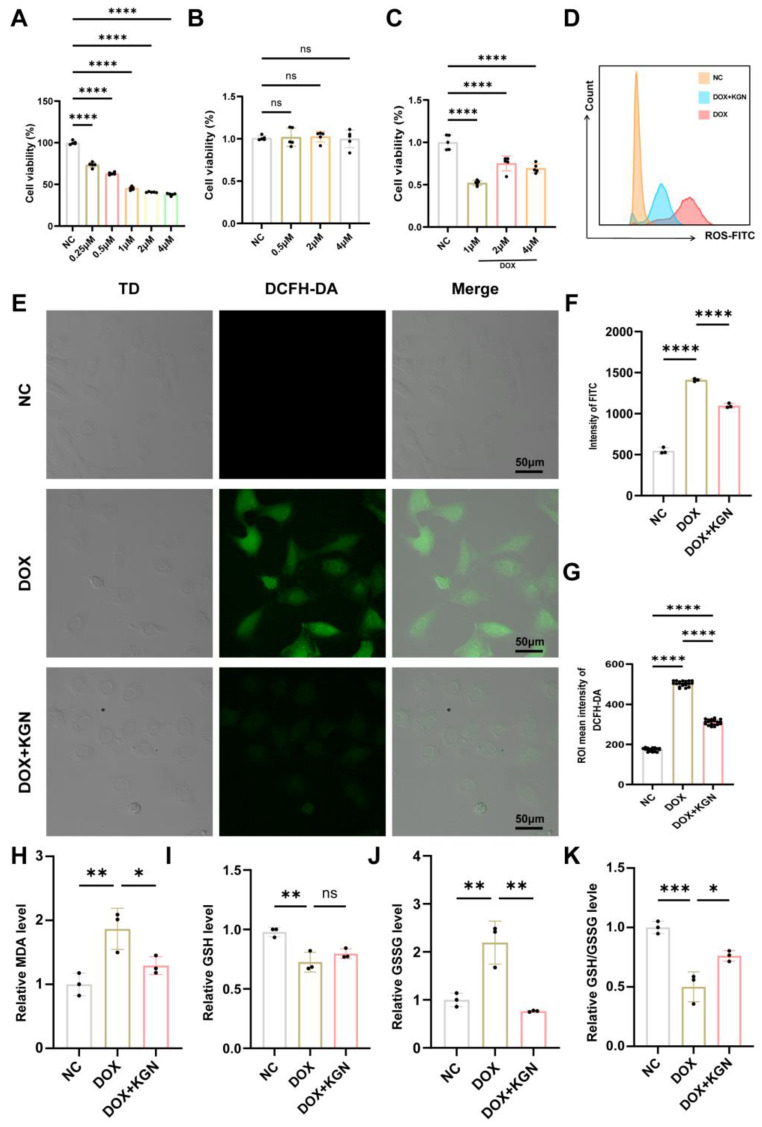
KGN treatment inhibited myocardial oxidative stress in H9C2 cells. (**A**) CCK-8 assay was used to determine cell viability in DOX-treated H9C2 cells (*n* = 5). (**B**) CCK-8 assay was used to determine cell viability in KGN-treated H9C2 cells (*n* = 5). (**C**) CCK-8 assay was used to determine cell viability of DOX combined with KGN-treated H9C2 cells (*n* = 5). (**D**,**F**) Flow cytometry analysis was conducted to assess cellular ROS levels (*n* = 3). (**E**,**G**) Immunofluorescence results of ROS (*n* = 20). (**H**–**K**) Relative levels of MDA, GSH, GSSG, and GSH/GSSG ratios in H9C2 cells (*n* = 3). All data were expressed as means ± SD. * *p* <0.05, ** *p* < 0.01, *** *p* < 0.001, **** *p* < 0.0001; ns means no significance.

To investigate the protective effects of KGN on DOX-induced inhibition in cardiomyocytes, we utilized H9C2 cells to act as an in vitro model. Consistent with previous studies, DOX treatment significantly reduced cell viability in a dose-dependent manner when compared to the PBS control group (Figure 4A). This highlights the cytotoxic nature of DOX at varying concentrations.

Subsequently, we treated H9C2 cells with different concentrations of KGN. Our results demonstrated that KGN alone did not affect cell proliferation at concentrations up to 4 μM, indicating that KGN is non-cytotoxic within this range (Figure 4B). Remarkably, when co-administered with DOX (1 μM), KGN significantly reversed the DOX-induced suppression of cell proliferation in a dose-dependent manner (Figure 4C). This suggests that KGN has a dose-sensitive protective effect against the antiproliferative impact of DOX on cardiomyocytes.

To further validate the antioxidative effects of KGN at the cellular level, we quantified intracellular ROS production, MDA levels, and the GSH/GSSG ratio in DOX-treated H9C2 cells. Consistent with our in vivo findings, KGN significantly mitigated the DOX-induced increase in oxidative stress markers. Specifically, KGN reduced ROS and MDA levels while enhancing GSH levels and restoring the GSH/GSSG ratio (Figure 4D–K). These results indicate that KGN effectively alleviates the redox imbalance and oxidative stress caused by DOX.

### 2.6. Mitochondrial Protective Effects of KGN in DOX-Induced Cardiotoxicity

The network pharmacology analysis and RNA-seq results highlighted the critical role of mitochondrial function in the therapeutic effects mediated by KGN. To further explore this, we examined mitochondrial changes in H9C2 cardiomyocytes subjected to DOX-induced stress.

**Figure 5:**
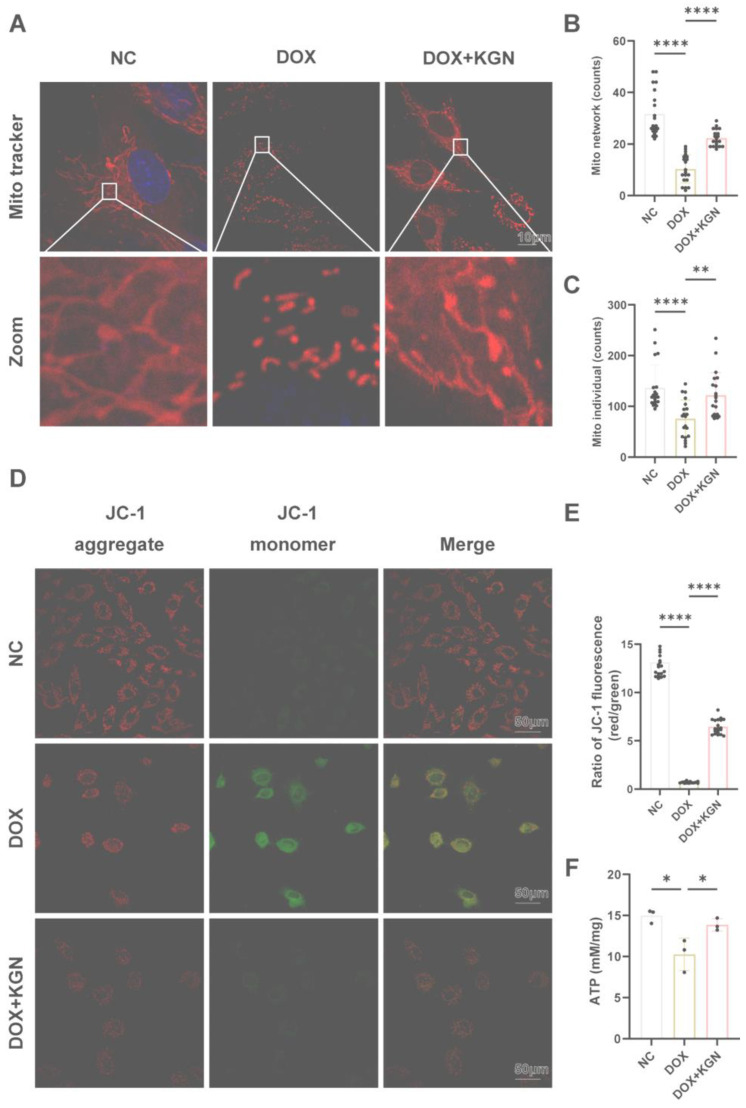
KGN protects the normal structure and function of mitochondria. (**A**–**C**) Mitochondrial structures in each group were identified through Mito tracker (red), with cell nuclei counterstained with Hoeshst (blue) (*n* = 20 in each group). (**D**,**E**) Effect of KGN on mitochondrial membrane potential reduction by DOX (*n* = 20 in each group). (**F**) Concentration of ATP (*n* = 3). All data were expressed as means ± SD. * *p* <0.05, ** *p* < 0.01, **** *p* < 0.0001.

DOX treatment led to a significant reduction in mitochondrial density and disrupted inter-mitochondrial communication. Under normal conditions, mitochondria exhibit a reticular, network-like structure essential for efficient cellular function. However, in the DOX-treated group, mitochondria appeared fragmented, shortened, and aggregated, losing their characteristic network architecture. This morphological alteration indicates severe mitochondrial dysfunction and compromised cellular energy dynamics. Remarkably, KGN treatment significantly reversed these changes (Figure 5A–C). Mitochondria in KGN-treated cells demonstrated restored density and reestablished their reticular network, indicating improved mitochondrial communication and integrity. Further functional assessment using the JC-1 assay revealed that DOX significantly decreased mitochondrial membrane potential, a hallmark of mitochondrial dysfunction. KGN treatment effectively restored this membrane potential, underscoring its role in maintaining mitochondrial health (Figure 5D,E). Additionally, DIC showed a notable reduction in ATP production, reflecting impaired mitochondrial bioenergetics (Figure 5F). KGN not only reversed this decline but also restored ATP levels, indicating the recovery of mitochondrial function and energy production. These findings suggest that KGN plays a critical role in preserving mitochondrial structure and function, counteracting the detrimental effects of DOX. These results indicate KGN could restore DIC-induced mitochondrial network integrity and enhance bioenergetics.

## 3. Discussion

Myocardial structural alterations and cardiac dysfunction are brought on by DOX treatment. This presents clinically as early asymptomatic left ventricular systolic impairment, which ultimately results in the development of refractory heart failure [17]. In this work, we showed that KGN reduced myocardial damage and cardiac insufficiency brought on by DOX, highlighting KGN’s dual protective efficacy against DOX-mediated oxidative insults and organelle dysfunction in particular. We verified that KGN might be a viable option for treating DOX-induced cardiotoxicity based on these data.

In this work, transthoracic echocardiography was used to evaluate heart function. The successful creation of our disease model was corroborated by the findings of Kuno et al., which showed that DOX lowered EF and FS and increased LVIDd and LVIDs in mice [18]. On the other hand, DOX-induced cardiac insufficiency was lessened by KGN. We discovered that KGN decreased the rise of cTnT in serum, which is a helpful marker for the early diagnosis of myocardial damage [19]. Furthermore, it was confirmed that KGN reduced DOX-induced myocardial damage by using HE and sirius red staining to look at tissue alterations. DIC has a complicated process, but Jeong et al. showed that enhancing antioxidant enzymes can effectively reduce DIC [20]. It has been demonstrated that KGN improves oxidative stress to protect disk degeneration. We therefore want to know whether KGN has the same antioxidant effect in DIC. To evaluate oxidative damage, we quantified MDA and GSH/GSSG ratio, a key indicator of antioxidant capacity, in both murine cardiac tissues and H9C2 cardiomyocytes. In line with our hypothesis, KGN treatment significantly attenuated DOX-induced elevations in MDA levels while restoring the GSH/GSSG balance compromised by DOX exposure. These data point to a mechanism by which KGN shields the heart from oxidative damage.

To delineate the cardioprotective mechanism of KGN against DIC, we implemented an integrated multi-omics strategy combining transcriptomic profiling with network pharmacology analysis. The results further confirmed that the mechanism by which KGN attenuates DIC is related to oxidative stress and mitochondria. Oxidative stress is characterized by a disruption of redox equilibrium within biological systems, marked by the excessive generation of ROS. These elevated ROS levels trigger oxidative modifications in essential cellular components, including protein structural alterations, the induction of lipid peroxidation cascades, and oxidative lesions in genetic material such as DNA [21,22,23]. KGN decreased the DOX-induced rise in ROS, according to our analysis of the ROS content in H9C2 cells using flow cytometry and DCFH-DA staining. We looked into how DOX affected mitochondria because of the strong connection between oxidative stress and mitochondria [24,25]. Yang et al. observed that enhancing mitochondrial function considerably reduced DOX-induced cardiac dysfunction [26]. In line with the results, our investigation revealed that DOX changed the morphology of the mitochondria in H9C2 cells. This could be because cardiolipin is only found in the mitochondrial membranes and is most prevalent in cardiomyocytes. However, by generating a nearly irreversible complex with cardiolipin that remains in the inner mitochondrial membranes, the cationic drug DOX, which has a high affinity for it, can interfere with the normal morphology of mitochondria [27]. In contrast, the addition of KGN treatment reduces the mitochondrial morphology disruption caused by DOX. The disruption of mitochondrial morphology affects its normal function. The production of ATP is one of the most important functions of mitochondria [28]. Consistent with our hypothesis, KGN treatment effectively prevented the decrease in intracellular ATP concentrations induced by DOX in H9C2 cardiomyocytes. Additionally, our investigation revealed that KGN administration attenuated the loss of mitochondrial membrane potential (ΔΨm) caused by DOX exposure. The maintenance of ΔΨm, which depends on the proton gradient across the inner mitochondrial membrane, is critical for supporting mitochondrial ATP synthesis through oxidative phosphorylation [29]. The disruption of this electrochemical gradient through mitochondrial impairment can compromise cellular viability by reducing both bioenergetic efficiency and ATP production capacity [30]. The mitochondria-targeted antioxidant mitoTEMPO has been reported to attenuate DOX-induced cardiac injury, and it was effective in maintaining the structural integrity of mitochondrial membranes and enhancing ATP production capacity [31]. Our findings demonstrated that the efficacy of KGN was almost the same as that of mitoTEMPO. Thus, we confirmed that KGN protects mitochondria and reduces DOX-induced mitochondrial damage.

Despite significant advances in understanding DOX-induced cardiomyopathy, including metformin’s autophagy-mediated cardioprotection and S-propylcysteine’s antioxidative effects, dexrazoxane remains the only US Food and Drug Administration (FDA)-approved therapy, albeit with concerning limitations such as myelosuppression exacerbation, antitumor efficacy interference, and secondary malignancy risks [32,33,34]. Our study proposes KGN—a compound renowned for its regenerative efficacy in cartilage repair, tendon healing, and wound regeneration, yet unexplored in cardioprotection—as a novel cardioprotective agent [35,36]. Crucially, KGN demonstrates a favorable safety profile with no reported severe adverse effects, addressing dexrazoxane’s critical drawbacks. While DOX cardiotoxicity involves multimodal pathomechanisms such as ferroptosis activation and IL-27p28-driven inflammation, our findings provide the first direct evidence that KGN alleviates cardiac damage through oxidative stress amelioration and mitochondrial preservation [37,38,39]. Although KGN’s inherent anti-inflammatory properties suggest potential multi-pathway efficacy, transcriptomic analysis indicates that its acute cardioprotection primarily stems from antioxidative and mitochondrial stabilization rather than canonical inflammatory pathway modulation [36]. Whether KGN acts in DIC through multiple pathways still requires further investigation. Looking forward, leveraging established KGN delivery systems, including cardiac-targeted nanoparticles and injectable hydrogels, could enable precise, sustained drug release to enhance therapeutic efficacy while minimizing systemic exposure [14,40]. Future studies should systematically investigate KGN’s chronic effects and combinatorial regimens with reduced-dose dexrazoxane to optimize clinical translation, building upon its unique mechanistic advantages and proven regenerative versatility.

Despite the novel findings of this study, several limitations should be acknowledged. First, the reliance on a single cellular model (H9C2 rat cardiomyocyte cell line) may restrict the generalizability of our conclusions. Although H9C2 cells are widely used for in vitro cardiac research, their immortalized nature may result in discrepancies in gene expression profiles and functional responses compared to primary cardiomyocytes under pathophysiological conditions. Future studies incorporating primary cells or human induced pluripotent stem cells (hiPSC)-derived cardiomyocytes could improve the physiological relevance of the findings. Second, the precise molecular targets underlying the observed intervention effects remain unidentified. While our data suggest potential pathway activation, the lack of direct mechanistic evidence limits the translational potential of this strategy. Further investigations utilizing proteomic profiling or genome-wide CRISPR screening are warranted to delineate the key mediators of the intervention.

## 4. Materials and Methods

### 4.1. Experimental Animal Models

Experimental protocols involving animal subjects were performed in compliance with the institutional guidelines established by the Laboratory Animal Ethics Committee of Chongqing Medical University Children’s Hospital (Ethical Approval Code: CHCMU-IACUC20241101010). Eight-week-old male C57BL/6J mice were sourced from HFK Biotechnology Co., Ltd. (Beijing, China) and maintained in a controlled environment with standardized photoperiod conditions (12 h light/dark cycle). The animals received unrestricted access to standard feed and autoclaved drinking water throughout the experimental period. A one-week acclimation period was provided prior to the initiation of experiments to ensure proper adaptation.

The study was designed with three experimental groups: negative control (NC), doxorubicin (DOX), and doxorubicin with KGN treatment (DOX + KGN), each consisting of ten mice. The experimental protocol involved the daily intraperitoneal administration of KGN (10 mg/kg/day) over seven consecutive days. Subsequently, mice were subjected to weekly intraperitoneal injections of DOX (4 mg/kg) or an equivalent volume of saline vehicle for four weeks. Following the DOX regimen, animals continued to receive either KGN (10 mg/kg/day) or saline for 7 additional days. At the conclusion of the treatment period, heart tissues were harvested for subsequent analyses.

### 4.2. Echocardiographic Assessment of Myocardial Function

The echocardiographic evaluation of myocardial function was conducted on day 7 following DOX administration. A two-dimensional guided M-mode echocardiography system (VisualSonics, Toronto, ON, Canada) was employed for the assessments. Mice were anesthetized using a continuous inhalation of 2–3% isoflurane to ensure minimal movement and stable heart rates during imaging. Monitoring comprised serial evaluations of ventricular morphology (LVIDs, LVIDd) and functional capacity (EF% and FS%) through standardized echocardiographic protocols. These parameters were automatically calculated using the integrated Vevo 3100 algorithms to ensure accuracy and consistency in the derived measurements.

### 4.3. Histological Examination and Staining

Cardiac specimens underwent immersion-fixation in 4% paraformaldehyde (PFA) followed by paraffin-embedding and serial sectioning at 5 μm thickness. Histomorphological evaluation via H&E staining combined by a quantitative histochemical approach using sirius red to assess interstitial collagen deposition. For fibrosis analysis, the percentage of sirius red-positive areas was calculated relative to the total field area to determine the collagen volume fraction. Imaging was performed with a Leica microscope (Leica Microsystems, Wetzlar, Germany), and ImageJ bundled with 64-bit Java 8 (National Institutes of Health, Bethesda, MD, USA) was utilized for the quantitative analysis of sirius red-stained sections.

### 4.4. Enzyme-Linked Immunosorbent Assay (ELISA)

The serum levels of cardiac troponin T (cTnT) were quantified using ELISA kits (Jiangsu Meibiao Biological Technology Co., Ltd., Yancheng, China) and according to the manufacturer’s protocol. Measurements were interpolated from a standard curve to ensure accurate quantification.

### 4.5. Cell Culture and Treatments

The H9C2 cell line (Procell Life Science and Technology Co., Ltd., Wuhan, China) was cultured in DMEM growth medium (Gibco, Grand Island, NY, USA) containing 10% fetal bovine serum, penicillin–streptomycin solution, and maintained under controlled atmospheric conditions (37 °C, 5% CO_2_).

### 4.6. Cell Viability

Cellular proliferative capacity was quantified via CCK-8 colorimetric assay (Beyotime, Shanghai, China). H9C2 cells were plated in 96-well microplates (3 × 10^3^ cells/well) for pharmacological interventions (DOX/KGN). Following 24 h pharmacological exposure, 10 µL CCK-8 reagent was introduced per well with subsequent 60 min chromogenic development. Metabolic activity was assayed by measuring optical density at 450 nm using a BioTek Cytation 5 multimode reader (Agilent Technologies, Santa Clara, CA, USA), with background subtraction using blank controls.

### 4.7. Measurement of GSH, GSSG, and MDA Levels

Oxidative stress parameters were quantitatively analyzed through the spectrophotometric determination of reduced GSH, GSSG, and MDA concentrations in both myocardial tissues and H9C2 cells. These assays were performed using commercial kits (Solarbio, Beijing, China), following the manufacturer’s instructions. Absorbance readings were obtained using a BioTek Cytation 5.

### 4.8. Flow Cytometry Analysis

Single-cell suspensions were generated via enzymatic dissociation utilizing a ROS assay kit. H9C2 cells were incubated with the ROS reagent (Beyotime, Shanghai, China) for 20 min at 37 °C. Flow cytometric analysis was performed on a BD FACSCanto™ II system (10-color configuration; BD Biosciences, Franklin Lakes, NJ, USA) with subsequent data processing using FlowJo.

### 4.9. Immunofluorescence Staining

H9C2 cardiomyocytes (3.0 × 10^5^/well) were cultured in confocal dishes and exposed to KGN for 2 h prior to 24 h incubation with 1 μM DOX. Cells were then stained with DCFH-DA (Beyotime, Shanghai, China), Hoechst 334342 (Beyotime, Shanghai, China), Mitochondrial probes MitoTrackerTM Red CMXRos (Beyotime, Shanghai, China) and JC-1 (Beyotime, Shanghai, China) was applied under optimized conditions following standard protocols. Post-staining, cellular samples underwent HBSS buffer washing and were subsequently imaged through Nikon A1R confocal microscopy (Nikon, Tokyo, Japan), with quantitative analysis conducted via NIS-Elements Viewer 5.21 64-bit (Nikon, Tokyo, Japan).

### 4.10. Detection of ATP Levels in Cardiomyocytes

Cellular ATP content was determined via commercial enzymatic assay (Solarbio, Beijing, China) following standardized protocols. Processed cells (1 × 10^4^/sample) underwent ice-cold lysis using probe sonicator (3 × 10 s pulses), followed by centrifugation (10,000× *g*, 10 min, 4 °C) to obtain clarified lysates. ATP quantification employed bioluminescent detection normalized against total protein concentration measured by BCA assay, ensuring data standardization across biological replicates.

### 4.11. Predict the Intersection of KGN Active Targets and DIC Disease Targets

Potential molecular targets of KGN were identified through integrated computational prediction using the PharmMapper and SwissTargetPrediction platforms. Disease-associated targets for DIC were systematically retrieved from GeneCards and OMIM databases through query optimization with standardized terminology. All gene identifiers were normalized to UniProt accession numbers for cross-database compatibility. Therapeutically relevant targets were subsequently determined through Venn diagram analysis of compound-target and disease-target networks.

### 4.12. Gene Ontology (GO) Enrichment Analysis

Functional annotation of KGN’s core targets in anti-DIC mechanisms was performed using the Database for Annotation, Visualization and Integrated Discovery (DAVID) bioinformatics database. Human genes were mapped through official gene symbols as primary identifiers. Statistically significant results (*p* < 0.05) were filtered and visualized using an interactive bioinformatics platform (Bioinformatics.com.cn), with pathway enrichment and molecular function analyses conducted through DAVID’s computational framework.

### 4.13. RNA Extraction, Library Preparation, and Sequencing

Total RNA was extracted from the experimental specimens with TRIzol Reagent (Thermo Fisher, Waltham, MA, USA) following standard operating procedures. To ensure genomic DNA removal, DNase I treatment (NEB, M0303L) was systematically implemented. RNA quality control included the dual evaluation of purity (A260/A280 ratios measured by Nanodrop OneC, Thermo Fisher, Waltham, MA, USA) and structural integrity (analyzed through LabChip GX Touch, PerkinElmer, Waltham, MA, USA). Quantitative RNA measurements were conducted using Qubit 3.0 fluorometry with the Broad Range RNA detection kit (Thermo Fisher, Waltham, MA, USA).

For sequencing library construction, the KCTM Digital mRNA Library Prep Kit (Seqhealth, Wuhan, China) was employed, featuring 12-nucleotide randomized unique molecular identifiers (UMIs) to mitigate PCR amplification artifacts and sequence duplication errors. Protocol optimization included size selection (200–500 bp fragments) through magnetic bead-based purification. Final library quantification preceded high-throughput sequencing on the DNBSEQ-T7 platform (MGI) using 150 bp paired-end configurations.

### 4.14. Statistical Analysisequencing

Statistical analyses were conducted using GraphPad Prism 9.0 (GraphPad Software, San Diego, CA, USA) with one-way ANOVA and Tukey’s post hoc tests. The results are presented as mean ± SD, with *p* <0.05 defining statistical significance.

## 5. Conclusions

These findings demonstrate that KGN mitigates DIC through dual mechanisms: attenuating oxidative damage and preserving mitochondrial integrity. In order to determine the exact chemical mechanism behind the action of KGN, more extensive studies must be conducted in the future.

## Data Availability

The original contributions presented in the study are included in the article; further inquiries can be directed to the corresponding author.
